# Assessment of Pharmacists’ Willingness to Conduct Medication Use Reviews in Poland

**DOI:** 10.3390/ijerph19031867

**Published:** 2022-02-07

**Authors:** Piotr Merks, Urszula Religioni, Magdalena Waszyk-Nowaczyk, Justyna Kaźmierczak, Artur Białoszewski, Eliza Blicharska, Anna Kowalczuk, Agnieszka Neumann-Podczaska

**Affiliations:** 1 Department of Pharmacology and Clinical Pharmacology, Faculty of Medicine, Collegium Medicum, Cardinal Stefan Wyszyński University in Warsaw, 01-938 Warsaw, Poland; piotrmerks@gmail.com; 2 Collegium of Business Administration, Warsaw School of Economics, 02-513 Warsaw, Poland; 3 School of Public Health, Centre of Postgraduate Medical Education of Warsaw, 01-826 Warsaw, Poland; 4 Department of Pharmaceutical Technology, Pharmacy Practice Division, Poznan University of Medical Sciences, 60-780 Poznan, Poland; mwaszyk@ump.edu.pl; 5 Zdrowit sp. z o.o., Pharmacy Chain, ul. Diamentowa 3, 41-940 Piekary Śląskie, Poland; j.kazmierczak@aptekizdrowit.pl; 6 Department of the Prevention of Environmental Hazards, Allergology and Immunology, Medical University of Warsaw, 02-091 Warsaw, Poland; abialoszewski@wum.edu.pl; 7 Department of Analytical Chemistry, Medical University of Lublin, Chodźki 4a St., 20-093 Lublin, Poland; elizablicharska@umlub.pl; 8 National Institute of Medicine, 00-725 Warsaw, Poland; a.kowalczuk@nil.gov.pl; 9 Chair and Department of Palliative Medicine, Poznan University of Medical Sciences, 61-245 Poznan, Poland; ar-n@wp.pl

**Keywords:** medication review, pharmacist, community pharmacy, role of pharmacists, readiness

## Abstract

Introduction. Pharmacists play an important role in healthcare. Their functions are evolving and, in many countries, they actively participate in interdisciplinary patient treatment. One of the most common services provided by pharmacists as part of pharmaceutical care in community pharmacies involves medication reviews. Objective. The objective of this study was to evaluate the readiness of pharmacists to conduct medication reviews in community pharmacies. Materials and methods. This study comprises 493 pharmacists from community pharmacies in Poland. A questionnaire (developed for the purposes of this study) was used. It consisted of eight questions regarding readiness to conduct medication reviews, along with personal data. Results. A total of 63.9% of the pharmacists were ready to conduct medication reviews, and 23.1% already had experience in this area. Participants were of the opinion that this service should be funded by the Ministry of Health or a third-party public payer, and overall was valued by the participants at PLN 169.04 (SD = 280.77) net per patient. Conclusions. Pharmacists in Poland have expressed their readiness to conduct medical reviews. Implementation of this service in community pharmacies in Poland can have a significant impact on optimising patient health outcomes.

## 1. Introduction

Throughout the years, the roles and perceptions of pharmacists in healthcare have evolved. Pharmacists not only dispense medicinal products, they also, in cooperation with therapeutic teams, have an active part in the patient treatment process [1,2,3]. Pharmacists, as one of the largest groups among medical professions, play an important role in public health in certain countries [1]. Studies show that the active participation of pharmacists in patient treatment, along with the development of pharmaceutical care, significantly improve patient health outcomes, compared to standard therapies [4,5]. Although in many countries the roles of pharmacists are much more developed [3], pharmacists in Poland have expressed their readiness to expand the scope of services they offer in community pharmacies to include, e.g., vaccinations [6]. This is particularly significant due to the potential of pharmacies in Poland (approximately 13,300 community pharmacies and 1300 pharmacy outlets, covering 57% of individual and 43% of chain pharmacies) [7], and the relatively large number of pharmacists (approximately 26,700 pharmacy masters and 1.85 pharmacists per pharmacy) [8]. In Poland, despite the large role of community pharmacies, the provision of pharmaceutical care services is not reimbursed, except for the reimbursement of the ex-tempore preparation (pharmacy compounding), and there are currently no publicly-funded pilots.

In 2021, government and science representatives prepared a report titled “Pharmaceutical Care” on the main assumptions of pharmaceutical care in Poland [9]. Pharmacist medication reviews or medicine use reviews (MUR) were considered the fundamental pillars of pharmaceutical care. A medication review is the primary tool of pharmaceutical care provided in pharmacies in Europe [10,11]. According to the definition of the Pharmaceutical Care Network Europe from 2018, a medication review is a structured evaluation of a patient’s medicines with the aim of optimising medicine use and improving health outcomes [12]. This entails detecting drug-related problems and recommending interventions.

A medication review is usually a structured interview with a patient, aimed at increasing patient knowledge about their medications and optimising their pharmacotherapy [13]. It can encompass medications administered by the patient, including those recommended by the doctor and used in the course of self-medication, as well as dietary supplements and foodstuffs intended for particular nutritional uses [14,15]. This situation is particularly important in the context of the lack of a general information system with complete patient medical records, which can lead to specialists lacking knowledge about products prescribed by other doctors. Medication reviews can play a significant role in the face of extensively used polypharmacy and self-medication [12,16,17], particularly among elderly people [18,19].

Studies to date indicate that the implementation of medication reviews allows detecting medication errors in patient treatments [20,21], which is of great importance when one considers that about 15.4% of hospital admissions are due to drug-related problems, and that almost half of them can be avoided [22,23]. Medication reviews also improve the disease management process and a patient’s quality of life, increase a patient’s knowledge on medications, and reduce the global costs of treatment [24,25].

Therefore, the objective of this study was to evaluate pharmacists’ readiness to conduct medication reviews in community pharmacies in Poland.

## 2. Materials and Methods

The study was carried out between 1 December 2020 and 28 February 2021, among pharmacists from community pharmacies in Poland, prior to the official announcement of a MUR pilot project run by the Ministry of Health in Poland. The research tool was an anonymous online questionnaire consisting of 8 questions regarding readiness to conduct medication reviews (experience in conducting medication reviews; medication reviews service plan; estimated time of medication reviews in contact with the patient; estimated preparation time for medication reviews; expectations as to the remuneration for the performance of a medication review; the institution that should pay for medication reviews; opinion on patient cooperation for a MUR; a proposal for a fee to be paid by the patient for a MUR), along with personal data (sex, age, specialisation, academic degree, job seniority, place of work). Our questions related to both simple and comprehensive (complex) medication reviews. For the purposes of the study, we considered a simple medicine review as a retrospective evaluation of treatment along with a short interview with a patient, and a comprehensive review consisted of information about medications, clinical data, and a long interview with a patient.

Our aim was to also try and assess the remuneration (for the performance of a medication review) that pharmacists would like to receive. This is of particular importance, taking into account that reimbursements of the drug review service are planned in Poland. Our goal was for pharmacists to set a remuneration that would reflect their workload for the performance of a MUR.

For reach respondent, we used direct contacts to organizations representing the Polish Pharmacy Trade Union (ZZPF). Emails were sent directly to pharmacist members via an email and through organisation Facebook and Twitter profile. The study was approved by the Bioethics Committee of the Nicolaus Copernicus University in Toruń at the Ludwik Rydygier Medical College in Bydgoszcz.

The results of the study were produced using descriptive statistics.

## 3. Results

### 3.1. Demographic Characteristics of the Respondents

The study participants involved 493 pharmacists, comprising 78.7% women and 21.3% men. The mean age of respondents was 36.17 years (SD = 8.11). The youngest respondent was 23 years old and the oldest respondent was 66 years old. Nearly every fourth respondent had a specialisation, of which pharmacy prevailed. Almost all of the respondents held a master’s degree in pharmacy. The mean job seniority was 11.07 years (SD = 8.21; minimum 0 years, maximum 43 years). The majority of pharmacies were located in cities, and the lowest number of pharmacies were in villages. Detailed data on the characteristics of the respondents are presented in Table 1.

### 3.2. Readiness to Conduct Medication Reviews

In total, 23.1% of the pharmacists reported experiences conducting medication reviews and 63.9% were ready to provide this service to patients (Table 2).

According to most of the respondents, medication reviews should be funded by a third-party payer (81.3%), or the Ministry of Health (61.5), the social insurance institution (21.1%), additional insurance (20.9), employers (14.0%), or patients (3%).

According to the pharmacists, if the cost of the medication review was to be borne by the patients, then the price should be about PLN^1^ (PLN exchange rate to USD and Euro as of 18 August, 2021: PLN 1 = USD 0.26; PLN 1 = EUR 0.22) 37.20 (SD = 45.45; minimum 0, maximum 400, mode 20).

While responding to the question: “What are your expectations regarding the remuneration for conducting a medication review? Please provide a net amount for 1 review, including 2 patient visits and an analysis of pharmacotherapy (in PLN)”, the pharmacists indicated that this remuneration should be about M = PLN 169.04 (SD = 280.77; minimum 5, maximum 5000, mode 100).

According to pharmacists, the average time needed to complete one medication review is 21.5 min during the first visit and 16.4 min during the second visit. For a comprehensive review, these values were 40.8 min and 30.5 min for the first and second visits, respectively (Table 3).

The estimated amount of time spent on preparing for a short medication review was valued at approximately 29.7 min during the first visit and 23 min during the second visit. In order to prepare for a comprehensive medication review, the pharmacists needed 53.3 min during the first visit and 41.9 min during the second visit (Table 4).

## 4. Discussion

Our study indicates that 63.9% of the pharmacists are ready to conduct medication reviews, and nearly every fourth respondent has experience in this area. Due to the lack of reimbursement of these services in Poland under the current law, pharmacists with experience in drug reviews could obtain them by participating in pilot projects.

The results are particularly important, taking into account the trust of Poles in pharmacists, as well as the openness toward providing pharmaceutical care services in community pharmacies [26]. The openness of Polish pharmacists is also of great importance, as also confirmed by other studies conducted in community pharmacies in Poland [6].

According to the plans, medication reviews conducted in Poland [9] will include an interview with a patient and a complete review of administered medications. Their objective will be to evaluate the validity of treatment, detect potential drug-related problems, and optimise treatment outcomes. It is recommended that elderly people at risk of polypharmacy (simultaneous administration of five or more medications) and patients with medical orders for reviews (e.g., after changing the course of treatment ordered by a doctor) should be automatically qualified for reviews.

To date, several pilot medication review programmes have been carried out in community pharmacies in Poland, including OF-Senior [27], where 291 medication reviews were conducted among patients aged ≥ 65 years with severe polypharmacy (administering ≥10 pharmaceutical products).

The respondents claimed that the total time required to prepare for a simple review (two visits) was approximately 53 min, and for a complex review—approximately 95 min, with less time spent on preparation for the second visit. The duration of a medication review in contact with a patient was estimated at about 38 min for a simple review (the total time of two visits), and 71 min for a complex review.

Taking into account the length of preparation and duration of MR, the scope of the medication review to be introduced in the first stage of implementation of this service in Poland should be carefully considered. It is also important to consider specific groups of patients who will be able to be referred to comprehensive MR services (e.g., the elderly, with multiple diseases). The scope of medication reviews should form the basis for further research and discussion in Poland.

Apart from checking the relevance of administered medications and detecting adverse effects, medication reviews can include suggestions for using (or stop using) dietary supplements, patient education regarding their treatments, and improving adherence. This is of particular importance in the situation of large patient access to medicines, mainly outside the pharmacy, which is characteristic in Poland [14]. Studies show that pharmaceutical services reduce the risk of potential adverse effects, improve patient treatment outcomes [28], the level of medication adherence [29], and the effects of therapy [30]. Moreover, medication reviews lead to rationalisation of treatment costs, which is of particular importance in the face of constantly growing expenses for healthcare, including medications and solving drug-related problems [25,31].

While conducting medication reviews, pharmacists have the opportunity to optimise expenses by suggesting changes to a treatment plan (e.g., when it is possible to switch from intravenous to oral therapy), indicating ceasing a specific medication if its administration can cause drug-related problems. Any of these situations will influence the costs and contribute to avoiding additional expenses in the event of adverse reactions [25]. As a result, medication reviews can prevent hospitalisation and the deterioration of patient health. Studies indicate that, even when hospitalised, more than 16% of patients can experience adverse effects [32], which extends hospital stays by two days on average and generates additional costs of USD 2000–2500 [32,33,34]. A systematic review of hospitalisations due to drug-related problems indicates that this percentage ranges between 1.3% and 41.3% (a mean of 15.4%). Medication reviews are of particular importance among elderly people, who, due to their multiple morbidities, are most vulnerable to polypharmacy [35]. For this reason, interviews with patients should include their preferences and possibilities, so that they could adhere to their therapies to the largest extent possible [36].

The lack of an unequivocal definition of a medication review, various models of this service, and different understandings of this concept by pharmacists, make it difficult to determine how many countries provide medication reviews [11,24,37].

An analysis of medication reviews in 34 countries in Europe indicates that they are conducted in more than half of them. Medication reviews vary between countries, to include services from retrospective evaluations of applied treatments (France, Norway, Holland), through the retrospective evaluation of treatments along with interviews with patients (Croatia, Great Britain, Sweden, Portugal, Spain, Switzerland), to comprehensive reviews consisting of information about medications, clinical data, and interviews with patients (Austria, Finland, Holland, Great Britain—services conducted outside pharmacies, mainly in medical centres). In some countries, medication reviews, as pharmaceutical service, have been conducted for about 20 years (Finland, Switzerland, Holland, Great Britain, Portugal, Sweden), while in other countries, this service was implemented relatively recently, e.g., Northern Ireland and Spain (2016). In many countries, there are pilot medication review projects (Austria, Belgium, Denmark, Slovakia, Ukraine; the German pilot programmes are most advanced) [11]. Previous studies showed a similar percentage of countries where pharmacists conducted medication reviews [38].

Remuneration for these services in these countries are diversified. In our study, the pharmacists propose that medication reviews be funded by the Ministry of Health or a third-party payer, and the average remuneration for a comprehensive medication review should be PLN 169.04 (EUR 35–40). In the case of a simple medication review in Switzerland, including an analysis of administered medications, the remuneration is funded by state institutions, while in Germany the project is financed by a private insurer. In all of the countries the amount of the remuneration for interviews with patients along with an evaluation of treatment is fixed (EUR 30–80) [11]. Considering the above, the expected remuneration for the medication review services in our study seems justified.

Studies on pharmacists’ readiness to conduct medication therapy management (MTM) show that pharmacists who received remuneration for this service reported significantly smaller barriers, compared to pharmacists who worked without additional remuneration [39].

A study in Belgium on the opinions of family doctors and pharmacists on the barriers and incentives for medication reviews suggests that the pharmacists described this service as time-consuming and reported problems with accessing patient data (mainly laboratory data), and with selecting appropriate medical information. The pharmacists indicated the need for relevant reimbursement for the service along with additional training. Family doctors positively rated the activities of pharmacists and emphasised the great value of this service for patients [38]. In this context, while implementing medication reviews in Poland, it is important that pharmacists have access to the medical records of patients, namely to their medications. In order to provide comprehensive pharmaceutical care, cooperation between the various groups of medical professions is necessary, particularly doctors and pharmacists [34,40,41].

## 5. Limitations

This study has several limitations. First of all, due to the lack of a standardised questionnaire, we created our own survey that was distributed online, which is why we do not know how many pharmacists received information about this study. The estimated number of pharmacists who received information about the study is 2310 (ZZPF observers).

We realise that pharmacists’ readiness to conduct medication reviews can be influenced by factors that were not included in our study, e.g., job satisfaction, workload, personal features, willingness of additional remuneration, etc. Additionally, other factors, such as the location of the pharmacy (big city, village, etc.), may have influenced the results.

## 6. Conclusions

Pharmacists in Poland have expressed their readiness to conduct medication reviews in community pharmacies and a number of them have experience in providing this service, which is why the implementation of medication reviews could be successful and result in detecting drug-related problems, optimising patient treatment, educating patients on treatment, and improving adherence. Nevertheless, further studies are required in order to determine possible barriers in the implementation of this service, and in the willingness of patients to use it.

## Figures and Tables

**Table 1:** Demographic characteristics of the respondents (N = 493).

Variable	N, %
**Sex**	
Woman	388, 78.7
Man	105, 21.3
**Specialisation**	
Yes	123, 24.9
No	370, 75.1
**Academic title**	
MPharm	481, 97.6
PhD Pharm	10, 2.0
PharmD	2, 0.4
**Place of work**	
Village	24, 4.9
City <50,000 inhabitants	118, 23.9
City 50,000–100,000 inhabitants	69, 14.0
City 100,000–500,000 inhabitants	131, 26.6
City >500,000 inhabitants	151, 30.6

**Table 2:** Readiness to conduct medication reviews (N = 493).

Question	N, %
**Do you have any experience in conducting medication reviews?**	
Yes	114, 23.1
No	379, 76.9
**Are you going to conduct medication reviews?**	
Yes	315, 63.9
No	14, 2.8
I don’t know	164, 33.3
**Who should pay for medication reviews (you can select more than one answer)**	
The National Health Institution (third-party payer)	401, 81.3
The Ministry of Health	303, 61.5
The social insurance institution	104, 21.1
Additional insurance	103, 20.9
Employer	69, 14.0
Patient	15, 3.0
**Do you think that patients should share payment for medication reviews?**	
Yes	166, 33.7
No	372, 66.3

**Table 3:** Estimated time spent on a medication review in minutes (in your opinion, how long should a medication review in a direct contact with a patient last?) (N = 493).

	M	SD	Minimum	Maximum	Median	Mode
**Visit 1 for a simple review**	21.5	12.2	2	60	20	20
**Visit 1 for a complex review**	40.8	22.9	2	180	30	30
**Visit 2 for a simple review**	16.4	10.3	2	90	15	15
**Visit 2 for a complex review**	30.5	19.3	2	180	30	30

**Table 4:** Estimated time spent on preparation for a medication review in minutes (please indicate the approximate time you can devote to prepare for a medication review) (N = 493).

	M	SD	Minimum	Maximum	Median	Mode
**Visit 1 for a simple review**	29.7	24.3	0	180	20	20
**Visit 1 for a complex review**	53.3	44.4	0	360	40	40
**Visit 2 for a simple review**	23.0	21.4	0	180	15	15
**Visit 2 for a complex review**	41.9	60.2	0	180	30	30

## Data Availability

All data are available from the corresponding author.
